# Association between executive function and excess weight in pre-school children

**DOI:** 10.1371/journal.pone.0275711

**Published:** 2022-10-10

**Authors:** Narueporn Likhitweerawong, Jiraporn Khorana, Nonglak Boonchooduang, Phichayut Phinyo, Jayanton Patumanond, Orawan Louthrenoo

**Affiliations:** 1 Department of Pediatrics, Division of Growth and Development, Faculty of Medicine, Chiang Mai University, Chiang Mai, Thailand; 2 Department of Surgery, Division of Pediatric Surgery, Faculty of Medicine, Chiang Mai University, Chiang Mai, Thailand; 3 Center of Clinical Epidemiology and Clinical Statistic, Faculty of Medicine, Chiang Mai University, Chiang Mai, Thailand; 4 Department of Surgery, Clinical Surgical Research Center, Faculty of Medicine, Chiang Mai University, Chiang Mai, Thailand; 5 Department of Family Medicine, Faculty of Medicine, Chiang Mai University, Chiang Mai, Thailand; Ajou University School of Medicine, REPUBLIC OF KOREA

## Abstract

The association between executive function and excess weight is becoming increasingly evident. However, the results of previous studies are still inconclusive, and there is a lack of evidence in early childhood. This study aims to examine the association between executive function, in terms of overall and subscales of executive function (e.g., inhibition, working memory, and shifting), and weight excess in preschoolers. A population-based cross-sectional study was conducted on children aged 2–5 years of age from public and private schools in Chiang Mai, Thailand. Participants’ weights and heights were measured and classified into three weight status groups (i.e., children with normal weight, overweight, and obesity groups). Executive function was assessed using the parent-report Behavior Rating Inventory of Executive Function-Preschool (BRIEF-P). Multivariable polynomial regression was performed to analyze the association between executive function and weight status. A total of 1,181 children were included in the study. After adjusting for confounders, impaired overall executive function significantly increased the probability of being overweight (odds ratio [OR] = 2.47; 95% confidence interval [CI] 1.33 to 4.56). A similar trend of association was also found between impaired inhibition and overweight status (OR = 2.33; 95%CI 1.11 to 4.90). Furthermore, poor working memory was associated with both overweight and obesity (OR = 1.87; 95%CI 1.09 to 3.20 and OR = 1.74; 95%CI 1.09 to 2.78, respectively). Our data suggest that deficits in executive function, particularly inhibition and working memory, are associated with weight excess in preschoolers. Early promotion of executive function may be needed at this developmental age to prevent unhealthy weight status.

## Introduction

Children and adolescents are increasingly facing the health problems of weight excess. The worldwide prevalence of overweight and obesity in young children has continuously increased over the past few decades, regardless of geography and culture [1]. In the conceptual understanding of obesity, it is a chronic metabolic disease that primarily involves increased calorie intake and decreased physical exercise. To date, the evidence is apparent that obesity is a multifactorial disease, originating not only from obesity-related behaviors but also influenced by genetics, environments, and gene-environment interactions [2]. Robust evidence has demonstrated several risk factors associated with weight excess in childhood, such as preterm, low birth weight, lack of breastfeeding, excessive screen time, and deficient amount of sleep [3–7]. Neurocognition, particularly executive function (EF), has been assumed to contribute to excess weight through the theoretical hypothesis of lacking control related to eating behaviors [8, 9].

EF involves the high-level mental processes required to control thoughts, emotions, and behaviors. Three fundamental EFs are inhibitory control/inhibition, working memory, and cognitive flexibility/shifting [10]. These three core EFs contribute to higher-order EF, such as problem-solving, reasoning, and planning [10]. EF develops dramatically in the preschool period [11], corresponding to the time of decline in body fat mass, before reaching the inflection point of adiposity rebound (adiposity rebound usually occurs about 6 years of age) [12]. The energy expenditure to develop the brain, especially the area of the brain related to EF, during this early childhood might influence the composition of body fat and the changes in body mass index (BMI) later [13, 14].

Several studies investigating the correlation between lower EF and higher BMI have focused mainly on school-age children, adolescents, and adults [15–18]. Most of the key results from these studies are found to be a small to medium effect size of the association. A paucity of studies regarding EF and weight excess has been conducted on preschool children due to the relatively low prevalence of overweight and obesity in preschoolers (whose body fat typically declines during this period) and the scarcity of valid instruments to measure EF in this age group [19]. Furthermore, the results of some studies conducted on young children are inconsistent. For example, Pieper and Laugero reported that BMI percentile was not correlated with most measures of EF in children 3–6 years old [20]. Tandon and colleagues showed that effortful control and delay ability were not associated with BMI changes [21]. Similarly, a recent study by Gross and colleagues reported that the EF score was not a predictor of weight status in preschoolers [22]. On the other hand, Hughes and colleagues indicated that eating self-regulation was related to the child’s BMI-for-age z-scores [23]. Schmitt and colleagues also suggested a negative correlation between the composite EF score and BMI percentile in preschoolers [24].

Due to a few studies focusing on young children and the inconsistent results mentioned above, this study aims to investigate the association between EF and weight excess in preschool-aged children by controlling for potential confounders reviewed from previous literature. The findings of this study may contribute to an understanding of the relationship between these variables in early childhood and obesity intervention by promoting EF.

## Materials and methods

### Study design and participants

This cross-sectional study was conducted on preschoolers from seven public and private schools in Chiang Mai, Thailand, between June to December 2021. Seven participating schools in Chiang Mai represented middle-class socioeconomic status (SES) based on the characteristics and facilities of the individual schools, such as the size of the school, number of students, teaching facilities, and school fees. The inclusion criteria were children aged 2–5 years and studying at the pre-kindergarten or kindergarten level. The exclusion criteria were 1) underweight children (BMI less than the 5^th^ percentile); 2) children diagnosed with neurodevelopmental disorders or genetic diseases reported by parents (e.g., autism spectrum disorder, attention-deficit/hyperactivity disorder, intellectual disability, and Down syndrome); 3) not being ethnically Thai; and 4) parents refusing to participate in the study. This study was approved by the Research Ethics Committee of the Faculty of Medicine, Chiang Mai University (051/2564). Before participating in the research, all parents/guardians of the child were informed of the relevant study detail. Their written informed consent was obtained.

### Measures

#### Study determinants–executive function

The parent-report version of the Behavior Rating Inventory of Executive Function–Preschool version (BRIEF-P) in Thai was used to measure EF of the child [25]. There were 63 items with a three-point Likert scale asking whether each behavior issue is problematic relative to other children of the same age, as never, sometimes, or often in the preceding six months. BRIEF-P measured overall EF (global executive composite) and EF subscales (i.e., inhibit, shift, emotional control, working memory, and plan/organize). The T-score for each scale was calculated based on standardized age and sex. Participants were categorized into impaired or normal EF groups, according to the cut-off point (T-score at or above 65), which was considered clinically significant regarding the difficulty of executive functioning [25]. This tool demonstrated good reliability with a Cronbach’s alpha of 0.80–0.95 for the EF subscales and global executive composite scale [25].

#### Study endpoints–weight status

The weight status was divided into three groups: 1) normal weight group was defined as preschoolers with BMI equal to or greater than the 5^th^ percentile but not reaching the 85^th^ percentile for children of the same age and sex; 2) overweight group was defined as preschoolers with BMI equal to or greater than the 85^th^ percentile but not reaching the 95^th^ percentile; 3) obesity group was defined as preschoolers with BMI equal to or greater than the 95^th^ percentile [26]. The body weight and height of the participants were measured at school using a digital weighing scale and stadiometer by teachers or school nurses. Then, the outcome assessors computed the percentile of BMI.

#### Pre-specified confounders

Potential confounders of overweight/obesity, including child’s age, sex, birth weight, gestational age, breastfeeding, maternal age, maternal BMI, maternal education, socioeconomic status, screen time, physical activity, sleep, and parenting styles, were reviewed from previous literature [3–7, 27–30]. Due to this substantial number of variables contributing to obesity and their potential complex interactions, we created a directed acyclic graph (DAG) using DAGitty [31] to determine confounding factors that require conditioning and to evaluate minimally sufficient adjustment sets of confounders when handling causal assumptions [32], as summarized in S1 Fig. A consensus was reached with all authors (i.e., developmental-behavioral pediatricians and clinical epidemiologists) to determine the conditioning and adjustments of confounders in DAG.

Age, sex, birth weight, gestational age, breastfeeding, maternal age, maternal BMI, maternal education, and screen time were evaluated using a general questionnaire. SES was assessed using the parent-report family income and classified into low (a monthly family income of not more than 18,000 Thai baht aTHB), and high SES (a monthly family income of over 85,000 THB) based on the Thai SES classification [33]. Physical activity and sleep questions were developed to assess the child’s duration of moderate to vigorous physical activity and sleep. These questions were validated and found to be acceptable in reliability and validity, as presented in S1 Table. Parenting styles were evaluated and classified into authoritative, authoritarian, and permissive styles based on Baumrind [34] using the Short Form of Parenting Styles and Dimensions Questionnaires (PSDQ)–Thai version [35]. PSDQ demonstrated good reliability and validity [35].

### Sample size

The sample size was calculated using the combination of ‘comparing two independent means’ and ‘rule of thumb of logistic regression’ methods, and then the largest sample size would be selected. Using the comparing two independent means method with alpha 0.05 (two-sided test), power 0.80, and effect size (mean and SD) from the previous study [24] required at least 500 samples, while using the rule of thumb of logistic regression needed at least 600 samples (overweight 60/obesity 60/normal weight 480) to evaluate six factors (5 EF subscales and 1 global executive composite scale), considering the prevalence of overweight and obesity approximately 10–20% [1, 36–38]. Finally, the total sample sizes of the overweight, obesity, and normal weight groups were at least 72, 72, and 576 individuals, respectively, considering an expected incomplete data/no response rate of 20%.

### Statistical analysis

Statistical analyses in this study were performed using Stata version 16 (StataCorp, College Station, Texas, USA) [39]. We used frequency (%), mean (SD), and median (IQR) to report descriptive statistics. To evaluate statistically significant differences between groups, the exact probability test and the one-way ANOVA test were performed for categorical data and normally distributed continuous data, respectively. The study endpoint “weight status” were considered as three discrete categories: overweight, obesity, and normal weight (a reference category) in the polynomial logistic regression model, rather than a range in the ordinal one. Although it was likely to have severity grading of weight excess (i.e., overweight and obesity), it could not be guaranteed that there is an association between the levels of the variables in this context of EF impacting weight excess. However, the goodness of fit of the two models was compared using the log-likelihood ratio test with no significant differences between the two models. Therefore, we selected the polynomial logistic regression as our representative model to assess the association between executive functioning and overweight/obesity in preschoolers. We also performed a multivariable polynomial logistic regression analysis separately for each EF subscale/global executive composite scale on weight status and reported the odds ratio estimates with 95% CI. We used a pairwise deletion technique to handle missing data. Statistical significance was defined as a p-value of less than 0.05.

## Results

### Baseline characteristics and comparing variables among individuals with overweight, obesity, and normal weight

**Fig 1:**
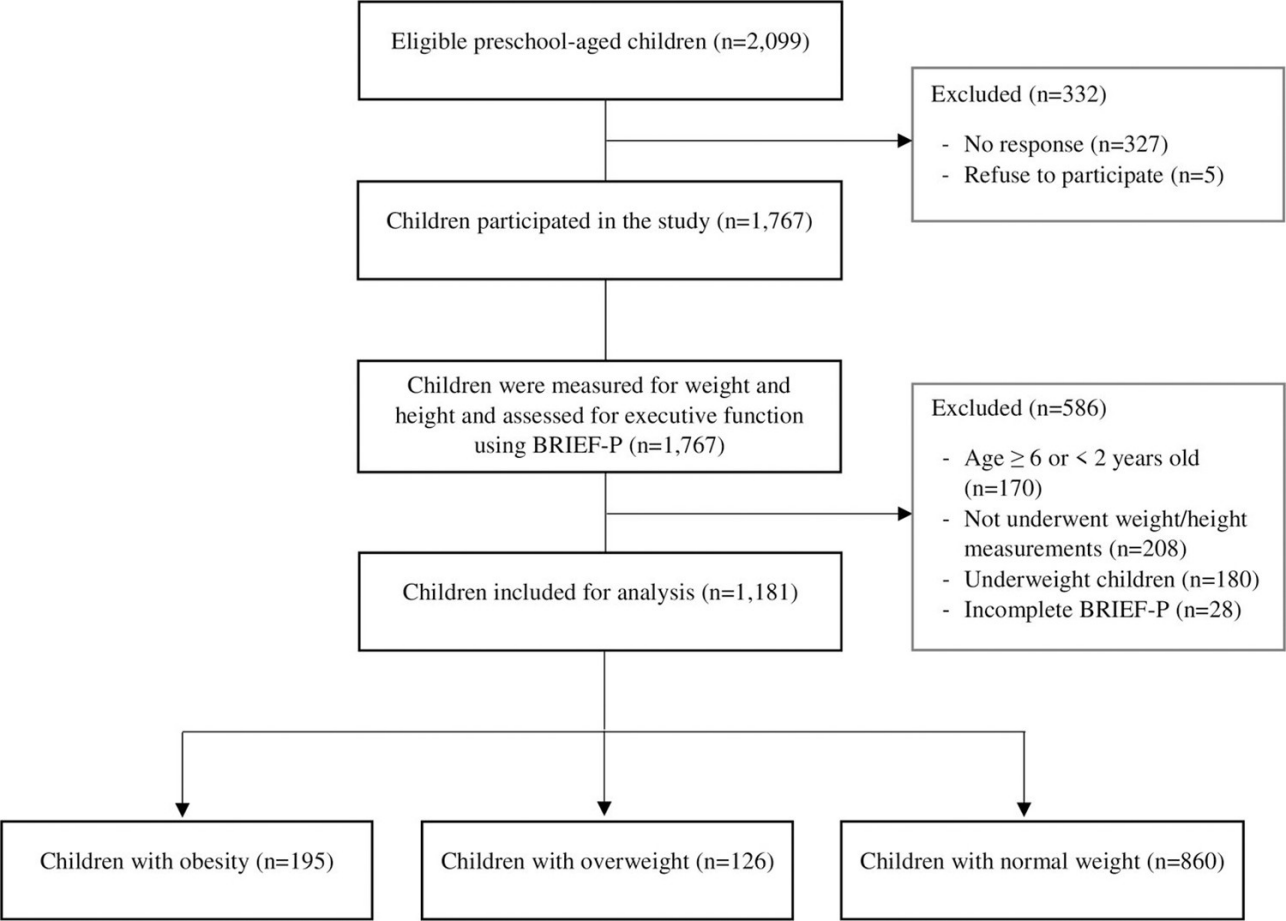
Study flow diagram.

A total of 1,181 healthy children were included in this study, as presented in the study flow diagram (Fig 1). The baseline characteristics are shown in Table 1. The potential variables contributing to weight excess categorized by weight status are presented in Table 2. The results show that *there* are significant differences in the variables of birth weight (p = 0.002), breastfeeding (p = 0.042), and maternal weight status (p < 0.001) across the three groups.

**Table 1:** General characteristics of the study participants.

Characteristic	No. of participants (Total N = 1,181)	Value
**Male, n (%)**	1,181	546 (46.23)
**Age (years), mean (SD)**	1,181	4.68 (0.85)
**Grade, n (%)**	1,181	
** Pre-kindergarten**		93 (7.87)
** Kindergarten-1**		260 (22.02)
** Kindergarten-2**		477 (40.39)
** Kindergarten-3**		351 (29.72)
**BMI, mean (SD)**	1,181	16.38 (2.48)
**BMI percentile, median (IQR)**	1,181	58.20 (29.80, 88.40)
**Weight status, n (%)**	1,181	
** Normal weight**		860 (72.82)
** Overweight**		126 (10.67)
** Obesity**		195 (16.51)
**Maternal age (years), mean (SD)**	1,157	35.53 (5.02)
**Maternal weight status, n (%)**	1,081	
** Normal weight**		816 (75.49)
** Overweight**		193 (17.85)
** Obesity**		72 (6.66)
**Maternal educational level, n (%)**	1,134	
** Lower than bachelor’s degree**		250 (22.05)
** Bachelor’s degree or higher**		884 (77.95)
**Socioeconomic status, n (%)**	1,125	
** Low**		174 (15.47)
** Middle**		831 (73.87)
** High**		120 (10.67)

Abbreviations: BMI: body mass index; SD: standard deviation; IQR: interquartile range.

**Table 2:** Possible variables contributing to weight excess categorized by weight status.

Variables	No. of participants (Total N = 1,181)	Obesity group (n = 195)	Overweight group (n = 126)	Normal weight group (n = 860)	p-value
**Male, n (%)**	1,181	99 (50.77)	61 (48.41)	386 (44.88)	0.289
**Age (years), mean (SD)**	1,181	4.78 (0.82)	4.77 (0.82)	4.64 (0.85)	0.052
**Gestational age, n (%)**	999				
** Preterm**		41 (23.98)	20 (17.70)	115 (16.08)	0.058
** Term**		130 (76.02)	93 (82.30)	600 (83.92)	
**Birth weight, n (%)**	1,043				
** Low birth weight**		20 (11.05)	7 (6.19)	54 (7.21)	0.002
** Normal birth weight**		145 (80.11)	97 (85.84)	671 (89.59)	
** High birth weight**		16 (8.84)	9 (7.96)	24 (3.20)	
**Breastfeeding, n (%)**	1,041				
** Never**		13 (7.47)	9 (8.11)	37 (4.89)	0.042
** No more than 6 months**		33 (18.97)	20 (18.02)	98 (12.96)	
** At least 6 months**		128 (73.56)	82 (73.87)	621 (82.14)	
**Moderate to vigorous physical activity, n (%)**	1,179				
** Less than 1 hour per day**		163 (83.59)	106 (84.13)	711 (82.87)	0.951
** At least 1 hour per day**		32 (16.41)	20 (15.87)	147 (17.13)	
**Screen use, n (%)**	1,171				
** More than 1 hour per day**		140 (72.92)	87 (70.73)	601 (70.21)	0.774
** Not more than 1 hour per day**		52 (27.08)	36 (29.27)	255 (39.79)	
**Sleep, n (%)**	1,176				
** Adequate amount for age**		151 (77.84)	98 (78.40)	715 (83.43)	0.097
** Inadequate amount for age**		43 (22.16)	27 (21.60)	142 (16.57)	
**Maternal age (years), mean (SD)**	1,157	35.82 (5.28)	35.89 (4.78)	35.41 (5.00)	0.419
**Maternal weight status, n (%)**	1,081				
** Normal weight**		103 (55.98)	88 (76.52)	625 (79.92)	<0.001
** Overweight**		59 (32.07)	20 (17.39)	114 (14.58)	
** Obesity**		22 (11.96)	7 (6.09)	43 (5.50)	
**Maternal education level, n (%)**	1,134				
** Lower than bachelor’s degree**		52 (27.96)	22 (18.49)	176 (21.23)	0.092
** Bachelor’s degree or higher**		134 (72.04)	97 (81.51)	653 (78.77)	
**Socioeconomic status, n (%)**	1,125				
** Low**		25 (13.23)	16 (13.11)	133 (16.34)	0.314
** Middle**		150 (79.37)	91 (74.59)	590 (72.48)	
** High**		14 (7.41)	15 (12.30)	91 (11.18)	
**Parenting styles, n (%)**	1,168				
** Authoritative**		187 (97.91)	123 (98.40)	826 (96.95)	0.973
** Authoritarian**		0 (0)	0 (0)	4 (0.47)	
** Permissive**		4 (2.09)	2 (1.60)	22 (2.58)	

Abbreviations: SD: standard deviation.

### Comparing executive function among individuals with overweight, obesity, and normal weight

**Fig 2:**
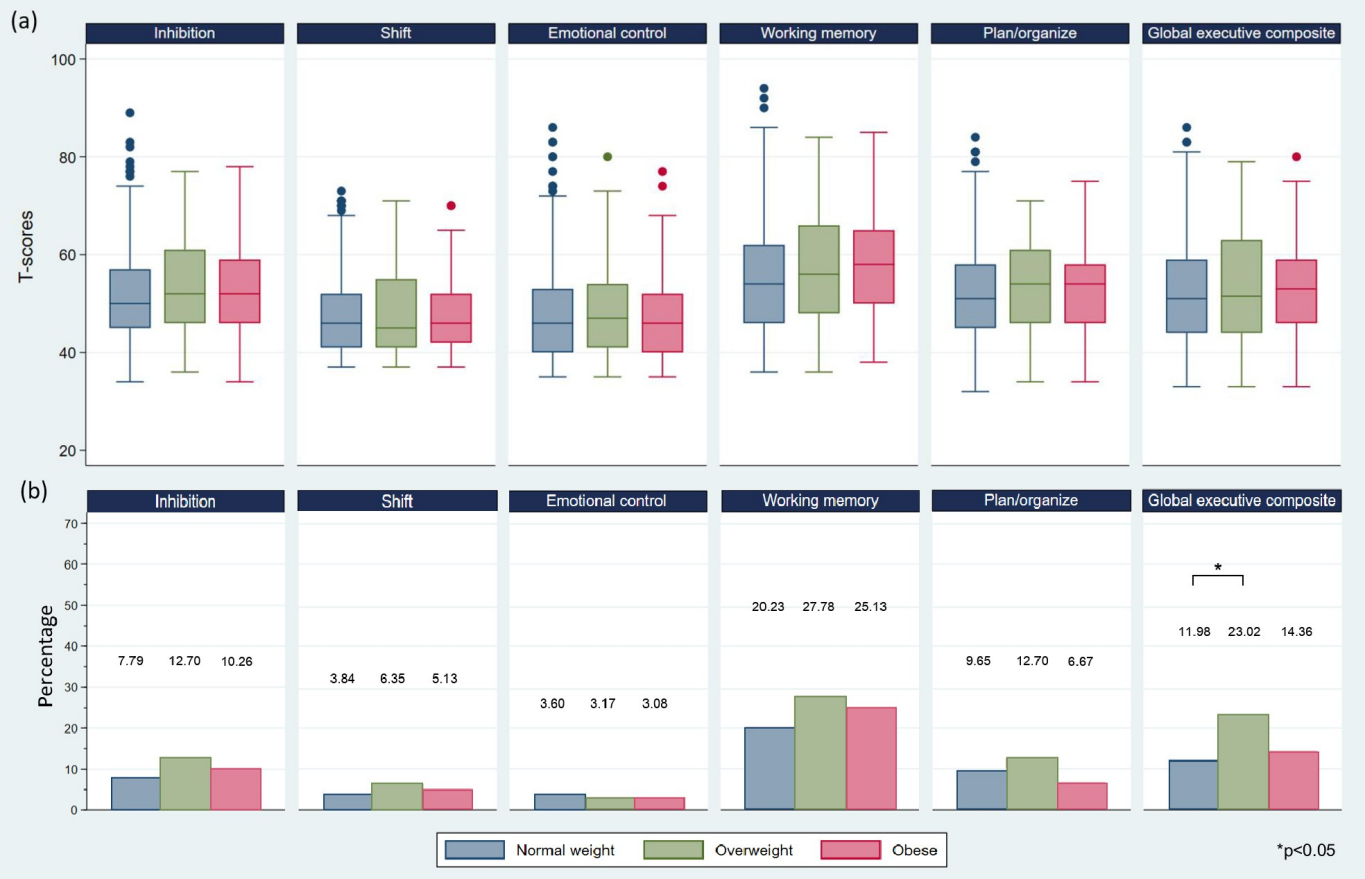
Boxplot and bar chart. The boxplot depicting T-scores of executive function subscales/global executive composite scale (a) and bar chart depicting the percentage of the number of children with impaired executive function among three weight status groups (b).

For EF T-scores, overweight and obesity groups have higher T-scores on all EF subscales and global executive composite scale compared to the normal weight group, as presented in Fig 2A. The result also shows that the working memory subscale has the highest T-score compared to the other EF subscales. No significant differences are found in T-scores between the three groups in most of the EF subscales and global executive composite scale, except for the marginally significant contribution of the working memory subscale (p = 0.045). After using a Bonferroni method for the multiple pairwise comparisons of the working memory subscale among the three groups, there are no significant differences in the T-scores between groups. When categorizing EF T-scores into EF status based on the cut-off point (impaired EF and normal EF), overweight and obesity groups show a higher percentage of participants with impaired EF than normal weight group on the global executive composite scale and most EF subscales, except for emotional control and plan/organize, as shown in Fig 2B. There is a significant difference only in the global executive composite scale (p = 0.005) between the three groups. After using the exact probability test for pairwise comparison for the global executive composite scale, the significant difference in the percentage of participants is found only in the normal weight and overweight groups (p = 0.001), while the other two comparisons (normal weight vs. obesity groups and overweight vs. obesity groups) do not show significant differences.

### Association between executive function and excess weight

The association between EF and excess weight using multivariable polynomial logistic regression (overweight vs. normal weight groups and obesity vs. normal weight groups) is presented in Table 3. After adjusting for confounders, the significant odds ratio observed in the polynomial logistic regression model (overweight vs. normal weight groups) indicates that individuals with impaired inhibition, working memory, and global EF are more likely to be overweight compared to those with normal EFs. The polynomial logistic regression model (obesity vs. normal weight groups) suggests that participants with impaired working memory are significantly associated with higher odds of being obese compared to those with normal working memory. The increased probability of being both overweight and obese is associated with impaired working memory. However, the effect of impaired working memory on overweight vs. normal weight does not significantly differ from the effect of impaired working memory on obesity vs. normal weight (X^2^ = 0.05, p = 0.832).

**Table 3:** Multivariable polynomial logistic regression analysis of weight status outcome and executive function subscales/global executive function scale^a^ (N = 799).

Executive Function	Obesity group^b^		Overweight group^b^	
	mOR^c^ (95% CI)	p-value	mOR^c^ (95% CI)	p-value
**Impaired inhibit**	1.84 (0.94–3.61)	0.077	2.33 (1.11–4.90)	0.026
**Impaired shift**	1.71 (0.66–4.45)	0.268	2.32 (0.86–6.22)	0.095
**Impaired emotional control**	0.60 (0.15–2.31)	0.457	0.80 (0.17–3.81)	0.777
**Impaired working memory**	1.74 (1.09–2.78)	0.019	1.87 (1.09–3.20)	0.023
**Impaired plan/organize**	0.66 (0.31–1.41)	0.280	1.07 (0.47–2.42)	0.866
**Impaired overall executive function (GEC)**	1.30 (0.73–2.34)	0.374	2.47 (1.33–4.56)	0.004

Abbreviations: mOR: multivariable polynomial logistic regression odds ratio; 95% CI: 95% confidence interval; GEC: global executive composite.

^a^ note that the multivariable polynomial logistic regression models were separately executed for each executive function subscales/global executive composite scale.

^b^ the reference group was the normal weight group.

^c^ adjusted for age, gender, gestational age, birth weight, breastfeeding, maternal age, maternal weight status, maternal education, socioeconomic status, parenting styles, screen use, moderate to vigorous intensity of physical activity, and sleep variables.

## Discussion

After adjusting for confounders using multivariable polynomial regression analysis, we found the associations between overall EF/EF subscales and excess weight. These associations were: i) overall EF was significantly related to the overweight status, but not obesity; ii) EF subscale–inhibition was significantly associated with overweight, but not obesity; and iii) EF subscale–working memory was significantly related to both overweight and obesity.

For the overall EF, this study was in line with previous studies that showed lower EF was significantly associated with weight excess [24, 40, 41]. Evidence shows that executive dysfunction can cause excess weight through unhealthy lifestyle behaviors such as increased high-calorie intake, decreased fruit/vegetable consumption, less physical activity, and more sedentary behaviors [17]. However, when we categorized excess weight into overweight and obesity separately, a significant association remained only in overweight. In our opinion, deficits in overall executive functioning in young children may not be a strong factor contributing to obesity, as seen in the previous study [41]. As we all know, additional factors contribute to obesity, such as genetics, eating behaviors, physical activity, and parenting and feeding styles. Most of the time, access to food and eating behaviors in young children depends on parental management of the food environment. Therefore, the EF of children may not have a direct and robust impact on their weight [22].

When considering the aspect of EF subscales, we found only inhibition and working memory related to excess weight. This finding was consistent with the previous study that showed a significant association between these EF subscales and weight excess [42, 43]. For EF subscale–inhibition, the possible link between inhibition and the risk of having excess weight is unhealthy eating behaviors. For example, children with lower inhibition usually have larger self-served portions and more food servings, display more compulsive overeating due to difficulties inhibiting response to food stimuli, and have less control to eat despite the absence of hunger [9, 23]. Subsequently, all these obesity-related eating behaviors lead to excess weight in later life. For EF subscale–working memory, working memory is the fundamental EF supporting self-regulation and inhibitory control by holding mental representation, cutting off distractions, and maintaining behaviors to achieve long-term goals [44, 45]. The explanation of the association between working memory and weight excess is that, in terms of maintaining a healthy weight, working memory helps engage with healthy habits and consequently leads to favorable physical health outcomes [46].

Unlike working memory, inhibition was significantly associated with overweight but not obesity. The undetectable association between inhibition and obesity in this study may reflect, to some extent, the clues to the direction of the association. Impaired inhibitory control could precede weight excess through overeating and obesity-related behavior mechanisms. Nonetheless, inhibition is not a sufficiently potent factor by itself to affect obesity, and hence it merely produces overweight. Conversely, working memory deficit could be a result from excessive weight via inflammation activity. Inflammatory responses from adipose tissue chronically induce brain alterations via neurohormonal transport perturbation, endothelial-neurovascular disruption, and ultimately changes in cortical gray matter and microstructure of white matter, which connect areas of the brain that facilitate working memory [47–49]. Therefore, once obesity presents, both overweight and obesity can impair working memory. However, there may be a bidirectional effect between these EFs and weight excess [15, 50]. Executive dysfunction may lead to a cycle of obesogenic behaviors and excess weight, which in turn induces inflammatory activity in the EF-related brain areas, resulting in impaired executive control over time [46].

We did not find a significant association between the other EF subscales (e.g., shifting and planning) and excess weight. Our finding contrasts with a previous study that reported shifting was significantly negatively correlated with the BMI percentile in preschool children [24]. This could be from the prior study that used performance tasks to assess EF, while our study used the parent-report BRIEF-P questionnaire. Another explanation is that a relatively low number of participants identified impaired shifting, which decreased the power to detect the significant association. The possible reason for the absence of significant relationships between planning and weight status is that planning is a higher-level EF that needs time to develop; thus, this ability at the younger age of our study is comparable among the normal weight and overweight/obesity groups.

The strength of our study is that we conducted a large sample size of typically developing preschool-aged children. We used DAG to manage the potential source of confounding for causal assumptions, which is an efficient and systematic method [51]. The findings found in this study support the body of knowledge in the prevention and early intervention of childhood obesity by promoting EF since early childhood. Emerging EF-targeted interventions such as computerized EF training and episodic future thinking, adjunct to the mainstay treatment of obesity, are mostly in preliminary studies but promising therapeutic paradigms to help promote EF and weight loss-related behaviors in children [52].

There are some limitations to this study. Firstly, we were unable to conclude the association as a causal relationship due to the cross-sectional nature of the design. Secondly, an indirect measure, such as BMI, may not be the best representative measure of obesity as body fat. However, BMI could be a good substitute for measuring obesity, as it is closely related to body fat, particularly in prepubescent children [53]. Thirdly, we were unable to examine all potential associated factors of weight excess, including unmeasured genetic effects that might confound the weight excess outcomes. Nonetheless, we collected maternal BMI and weight status as surrogate variables of genetic influence. Fourthly, there were a large number of sample exclusions; however, this study yielded sufficient power to detect the differences in executive functioning between weight status groups. Lastly, there could be benefits and weaknesses due to the homogeneity of the middle-class SES samples included in this study. This sample homogeneity could facilitate controlling covariates between individuals as they shared common ethnic and cultural behaviors, but it might limit the generalizability of the study to the low-/high-class SES populations.

In conclusion, this study found an association between deficits in EF and weight excess in preschoolers. The result suggests that inhibition and working memory might be key components of such a relationship. Strategies directly targeting the promotion of EF, specifically inhibition and working memory, in early childhood might help young children maintain a healthy weight throughout their developmental period. However, longitudinal studies are needed to investigate further the causal effect between EF and weight excess, and the treatment effect of EF intervention on weight outcomes.

## Supporting information

S1 Fig Directed acyclic graph (DAG). DAG presents the causal model of the association between executive function and overweight/obesity. Thirteen confounders (ancestor of exposure and outcome) were determined as the minimal sufficient adjustment sets for estimating the effect of executive function on overweight/obesity.
(TIF)

S1 Table Reliability and validity of the study questions related to physical activity and sleep. (PDF)
