# Supplementary figures and images for: Association between executive function and excess weight in pre-school children
Source: PLoS One. 2022 Oct 10;17(10):e0275711. doi: 10.1371/journal.pone.0275711 (PMC9550082; doi:10.1371/journal.pone.0275711)

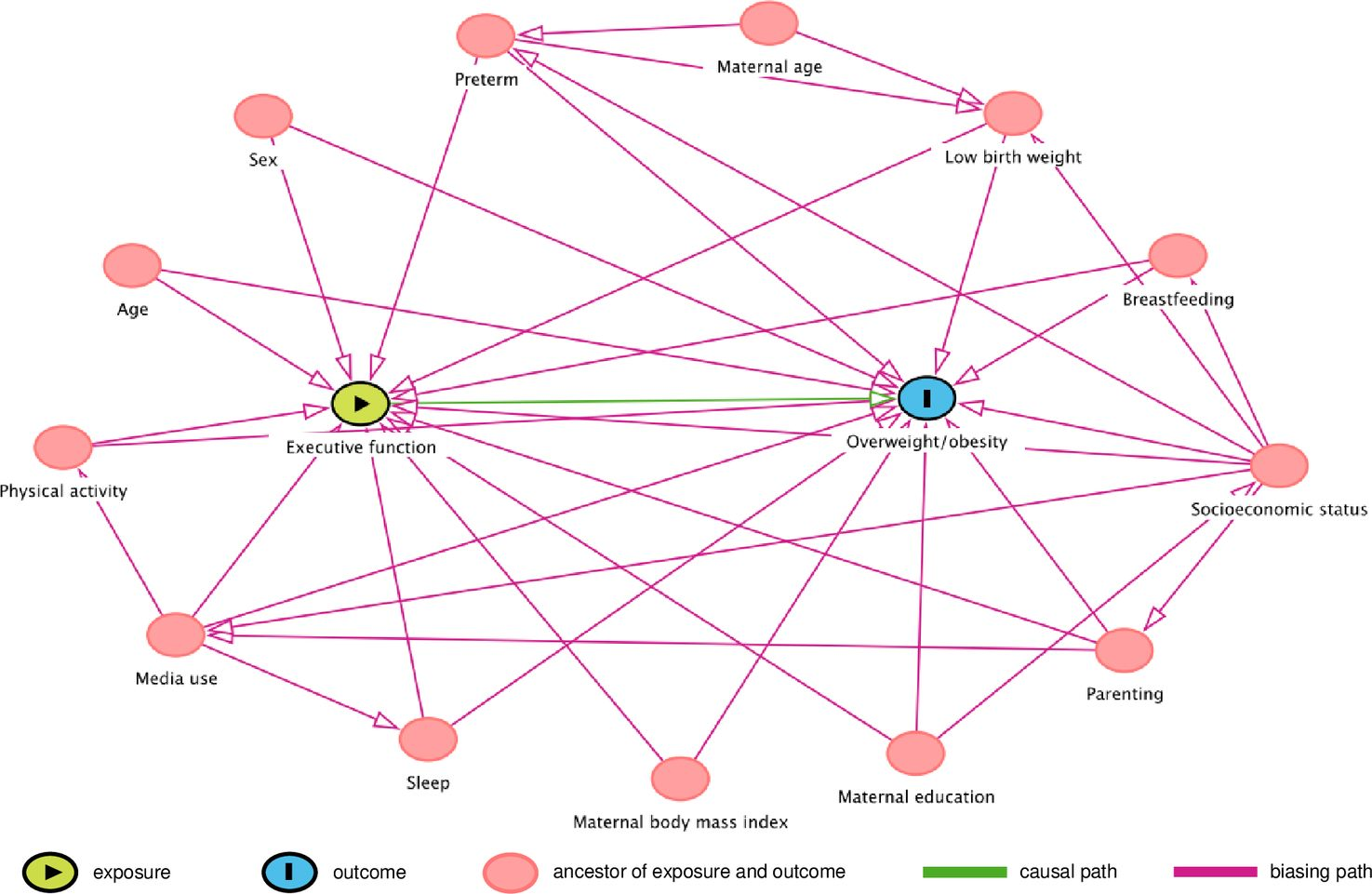

Supplement: S1 Fig — DAG presents the causal model of the association between executive function and overweight/obesity. Thirteen confounders (ancestor of exposure and outcome) were determined as the minimal sufficient adjustment sets for estimating the effect of executive function on overweight/obesity. (TIF) [file pone.0275711.s001.tif]
