# Supplementary material for: Association between executive function and excess weight in pre-school children
Source: PLoS One. 2022 Oct 10;17(10):e0275711. doi: 10.1371/journal.pone.0275711 (PMC9550082; doi:10.1371/journal.pone.0275711)
Supplement: S1 Table — (PDF) [file pone.0275711.s002.pdf]

S1 Table 1. Reliability and validity of the study questions related to physical activity and sleep.

Questions	Inter-rater reliability[a]	Instrumental validity[b]
**Physical activity**<br>**(1) On weekdays, how many days does your child engage in energetic play/exercise such as running and jumping or playing on a playground, ball games, swimming, and riding a bike?**<br>**The report options were 0 day; 1 day; 2 days; 3 days; 4 days; and all 5 days**<br>**(2) On weekdays, how long does your child engage in these activities accumulated throughout the day?**<br>**Free text report for minutes per day**<br>**(3) On weekends, how many days does your child engage in energetic play/exercise such as running and jumping or playing on a playground, ball games, swimming, and riding a bike?**<br>**The report options were 0 day; 1 day; all 2 days**<br>**(4) On weekends, how long does your child engage in these activities accumulated throughout the day?**<br>**Free text report for minutes per day**	0.74	0.62
**Sleep duration**<br>**(1) On weekdays, at what time does your child usually sleep at night and wake up in the morning?**<br>**Free text report for the time the child sleeps and wakes up**<br>**(2) On weekends, at what time does your child usually sleep at night and wake up in the morning?**<br>**Free text report for the time the child sleeps and wakes up**	0.78	0.62

[a] Inter-rater reliability was evaluated in 10 mother-father dyads primarily caring for the child. The mother and father were given a physical activity question separately. The average daily duration of moderate to vigorous physical activity (MVPA) reported by parents was classified as adequate (at least 60 minutes per day) or inadequate exposure to MVPA. The agreement of the child's MVPA exposure question from both parents was assessed using Cohen's kappa coefficient. The inter-rater reliability of the sleep question was also evaluated with the same method of MVPA.

[b] Instrumental validity, it was evaluated in 10 preschooler-mother dyads. The parent was given informed consent and the child was willing to wear a smart band that continuously monitored heart rate for one week. The MVPA period in one day was extracted from the wearable device for the duration of increased heart rate for age and sex. At a time of increased heart rate was accompanied by the logbook in which the mother noting the exact time the child participated in energetic play/exercise to ensure that the increased heart rate was from MVPA. The average daily duration the child participated in MVPA retrieved from the device was classified as adequate (at least 60 minutes per day) or inadequate exposure to MVPA. The results of a parent report and smart band were analyzed for the instrumental validity of the MVPA question using Cohen's kappa coefficient. The validity of the sleep question was also evaluated using the same method of MVPA.
